# The Impact of Children’s Food Neophobia on Meal Perception, Emotional Responses, and Food Waste in Italian Primary School Canteens

**DOI:** 10.3390/foods14101777

**Published:** 2025-05-16

**Authors:** Maria Piochi, Michele Antonio Fino, Luisa Torri

**Affiliations:** University of Gastronomic Sciences, Piazza Vittorio Emanuele II, 9, 12060 Pollenzo, Italy; m.fino@unisg.it (M.A.F.); l.torri@unisg.it (L.T.)

**Keywords:** food neophobia, mealtime, liking, emotion, waste, catering, context, school

## Abstract

Food neophobia (FN) has been poorly explored in real contexts and in large-scale studies with children. This study assessed the impact of FN in children on school canteen meals by considering liking, emotional status, and food waste behaviours. We involved 630 children (7–11 years old; females = 53%) from nine Italian primary schools. The main self-reported variables that were collected included pleasure of eating in the canteen, declared liking for different foods, emotional responses, meal description, and food waste. The characteristics of low neophobia (LN), medium neophobia (MN), and high neophobia (HN) were comparable between genders and provenience and did not differ by the pleasure of eating at home. Children with HN had the lowest frequency of eating in the canteen, the highest self-reported amount of wasted food, and the lowest liking for all items, especially vegetables and legumes; they selected mostly emotions with negative valence and described the meal as more uncomfortable and boring. Instead, children exhibiting LN used positive emotions with high arousal to describe the meal and found it a little boring, while those with MN showed an intermediate attitude. Children with HN may benefit from familiarisation actions to accept non-domestic meals and reduce food waste in non-familiar environments. Improving school canteen contexts (e.g., the socialising possibility) can modulate children’s emotional responses and reduce food waste.

## 1. Introduction

Food neophobia (FN) is a psychological construct explained as the reluctance to eat and/or the avoidance of novel foods [1] and is linked to many complex biological, psychological, and environmental factors. FN belongs to a wider group of eating disorders, i.e., sensory food aversions [2], and has been greatly studied over the last thirty years; a recent review notably showed that FN tends to reduce over time across countries due to globalisation [3]. FN is usually described as occurring at 20 months of age, although neophobic responses to tastes can be observed from birth [4]. Generally, however, FN is reported to change across the life course, being absent at birth, reaching a peak between 2 and 5 years, decreasing during adulthood, and probably increasing again in older age [5]. A large-scale study on the Italian population among adults showed significant effects of gender and age [6], reporting that males are more neophobic than females. However, most studies on the impact of gender on FN show no differences between males and females (see [7] and [3] for a review).

FN has been primarily and extensively studied in children [8,9]. Related factors affecting FN in children were reviewed in [10], with most studies on FN in children concentrating on Europe [11].

FN is associated with poor dietary variety and nutrient intake in children [11]. Indeed, the promotion of children’s healthy eating is a key public health priority in an international framework in which health is increasingly considered a broad concept under the umbrella of ‘One Health’ [12].

Moreover, FN in children is associated with the parental and family environment, showing a positive association with a chaotic and coercive parenting style [13] and a negative association with parents’ degree of education [9]. Since eating habits are formed by early childhood experiences of being in contact with foods and observing the environment [14], it is worthwhile studying FN through field studies in real consumption contexts that offer socialising situations (moments in which children can talk to and interact with each other). In fact, these contexts crucially allow us to highlight food choices and estimate real food behaviours.

Among the possible real contexts, school canteens are indeed among the primary environments worth studying due to their relevance in children’s everyday life and their educational valence. Importantly, despite considerable interest in FN in recent decades, large-scale studies exploring the impact of FN in children in school canteens are still scarce. Apart from methodological aspects, this is due to practical issues that hinder the effective activation of projects involving families and schools, such as difficulty in logistics, lack of time and available dedicated staff, and the length of ethical and legal management and fulfilment.

Children’s school meal acceptance is complex and depends on the interaction between the dish, student, and context intended as the environment [15]. Moreover, eating in the canteen implies social dynamics that develop during the meal that affect children’s emotional responses. For example, children who feel uncomfortable or worried about the environment tend to experience the meal as stressful. Emotional status can thus affect the amount of food eaten and the food waste. Despite the many studies published on emotions among young populations [16,17,18], to the best of our knowledge, the emotional status evocated by the canteens intended as complex spaces is little explored [16], with the impact of emotional status on food waste in socialising contexts even less explored [19].

In addition, food waste in school canteens, i.e., food left on plates after a meal, has been addressed in many studies considering consumer behaviours [20], but the literature is fragmented when considering the relationship between emotional status and important variables such as liking, which are used in consumer science to monitor children’s behaviours [19]. Indeed, reducing food waste across food systems, including collective catering, is one of the most urgent issues in food management. Importantly, while some studies have focused on the exploration of FN in children in school canteens [21], to the best of our knowledge, FN in children has not been studied in relation to the emotions aroused by food in that context nor in respect to food waste, namely the residual food left on plates after a meal in a school canteen.

In the existing literature, FN has been studied using validated tools in children aged 4–5 years participating in a series of test lunch meals at weekly intervals at school [22]; in 8- and 11-year-old Finnish children in relation to parental education [9]; and in 6–13-year-old children in relation to oral tactile sensitivity [23]. Tools to evaluate FN were recently reviewed [11]. Among these tools, the Italian Child Food Neophobia Scale (ICFNS) was specifically designed for children and reliably used with 7- and 8-year-old children [8]. The ICFNS has been successfully used in different European countries (Finland, Italy, Spain, Sweden, and UK) [24].

Considering the aforementioned gaps in the literature, the aim of this study was to explore the relationships between FN (evaluated with the ICFNS validated tool by Laureati and colleagues [8]) and variables describing children’s perceptions of mealtimes in school canteens, the pleasure of eating in the canteen, liking different food categories, emotional responses, meal description, and food waste. It is relevant that such relationships be studied simultaneously, since only two of these variables have been previously explored at the same time. Our hypotheses were as follows: I. highly neophobic children exhibit a more negative emotional status and negative meal description towards eating in the school canteen; II. the self-reported level of food waste is consequently negatively affected (higher food waste in highly neophobic children than in those with low neophobia).

## 2. Materials and Methods

### 2.1. School Selection

Nine Italian primary schools distributed in the national territory were invited to join the project described in this study (Milano—northwest; Cinisello Balsamo, Milan—northwest; Legnago, Verona—northeast; Formigine, Modena—northeast; San Marcello, Ancona—centre; Florence—centre; Perugia—centre; Ladispoli, Roma—centre; Salerno—south). School selection was conducted according to the following two criteria: being representative of existing Italian school canteen realities and being available to involve all classes, thus covering all age ranges (from first class to fifth class). For the first criterion, the selection of regions and the location of schools reflected the distribution of school lunch services in Italy, which are notably more prevalent in the northern regions of the country than in the south. Lombardy, in particular, has the distinction of being the most heavily served region in Italy (Openpolis report, 2021, available online: https://www.openpolis.it/servono-mense-scolastiche-dove-e-piu-diffusa-la-poverta-alimentare, accessed on 30 April 2025). Moreover, the selection aimed to mirror the demographic diversity of the nation, encompassing both prominent and less populous centres. For this purpose, major population centres such as the provincial capitals were included (Milan, Florence, Salerno, and Perugia), along with smaller centres in the provinces of Milan, Verona, Modena, and Rome. Data were collected between March and November 2022 (9 months). Each school autonomously established one moment of data collection in each class coordinated by teachers and supervised by researchers. Each class had a dedicated one-day session for data collection.

### 2.2. Children

Children aged from 7 to 11 years were involved, attending from the third to the fifth levels. Children from the first and second level were excluded from this part of the study on FN, since it is known from a large-scale study on the evolution of FN during the lifespan that a peak in FN is reached at approximately 5–6 years old [5], so we wanted to exclude the most acute phase of FN expression and thus include the most mature children. The children involved were able to read and write. The survey was delivered in Italian, and if needed, children (all being able to read and write) were assisted by teachers. Teachers were provided training via an online training session with experimenters before the test. A written instruction prepared by the experimenters was shared with all schools before the test to standardise the pre-test explanation delivered by teachers. After the questionnaire completion, the general exclusion criteria were (1) not having answered one of the essential questions on gender, class, liking for meal and items, or meal description choice (these variables were included here for their essentiality; other single missing values were admitted) and (2) having incongruency in responses within a variable (e.g., indicating that one attended two different levels). After excluding 14 respondents who did not meet the inclusion criteria, 630 children were retained for data analysis. The sample was a convenience sample, and FN was determined after data collection.

An informed consent form was signed by a family caregiver (or a legal guardian) for each child. The study followed the Declaration of Helsinki, and approval was obtained from the Ethical Committee of the University of Gastronomic Sciences (Ethical Committee Proceeding N. 2022.03).

### 2.3. Experimental Plan and Questionnaires

The procedure adopted in this study is part of a wider research project explained in detail in one of our recently published works [19]. However, this study specifically focuses on a subset of data (food neophobia) that has not previously been presented. Briefly, a paper questionnaire was distributed in each class with the assistance of teachers after a teacher training phase was conducted online. Some trials were run at schools with children to check the degree of comprehensibility of the questionnaire before the test. To reduce variability in instructions provided by teachers, written instructions were prepared by the experimenters and shared with all schools before the test. The whole dataset included socio-demographic variables (age, gender, country of birth: nationality), the frequency of eating at the school canteen, the pleasure of eating in the canteen and at home (How much do you dislike/like eaten in the school canteen/home? 5-point scale: 1 = per niente = not at all; 3 = così così = neither dislike nor like; 5 = moltissimo = extremely like). In addition, it included the following core variables described below: declared liking for different foods, a description of the mealtime in the canteen considering environmental aspects, food waste, and emotional responses. In particular, children’s liking for seven food categories eaten in the canteen was collected (pasta; rice; vegetables; beans—peas, lentils, etc.; fruits; meat; fish) using an adapted version of the Italian validated scale with children of the same age [8] (How much do you dislike/like these foods eaten in the school canteen? 5-point scale: 1 = cattivissimo = extremely dislike; 2 = cattivo = dislike; 3 = così così = neither dislike nor like; 4 = buono = like; 5 = buonissimo = extremely like). The seven food categories were chosen for being highly spread in any Italian region. Children were asked about their overall food waste after lunch in the canteen (How much food remains on your plate after lunch in the canteen? 1 = no residual food on my plate; 6 = all food was left on my plate) and food waste for specific food categories eaten in the canteen (Which foods mostly remain in your plate after lunch in the canteen? Select all items that you want: pasta; rice; vegetables; beans—peas, lentils, etc.; fruits; meat; fish). Children were asked to describe the meal (How would you describe the lunch in the canteen? (Only 1 choice per pair)) considering the following: duration (short/long), presence of noise (noisy/silent), perceived comfort (comfortable/uncomfortable), crowding (crowded/empty), fun (funny/boring), and the possibility of movement (free to move/not free to move). The emotional status associated with a meal in the canteen was collected with a check-all-that-apply (CATA) test on nine emotions (How do you feel eating in the canteen?). Emotions, verbally described and associated with emoticons, were selected based on the previous literature [17,18]: one emoticon for neutral emotional status, four emoticons for positive emotions (happy, satisfied, enthusiastic, full of energy), and four emoticons for negative emotions (sad, annoyed, angry, disgusted). Finally, the validated Italian Child Food Neophobia Scale (ICFNS) was adopted [8]; this scale consists of 8 items (4 neophobic and 4 neophilic) and a 5-point scale (1 = falsissimo = very false for me; 2 = falso = false for me; 3 = così così = so-so; 4 = vero = true for me; 5 = verissimo = very true for me). This stage (ICFNS) was the only one administered after lunch in order to minimise the boredom due to due to the length of the questionnaire, while the entire first part of the questionnaire was administered before lunch. Between the first and the second questionnaire, approximately 60–90 min passed. The fulfilment of the two questionnaires lasted approximately 20–40 min.

### 2.4. Data Analysis

Mean values are reported in the text associated with the standard error of mean and, where relevant, with letters from Tukey’s test (*p* < 0.05) applied after performing an analysis of variance (ANOVA). Schools were classified according to the Italian geographical classification (NUTS areas) [25]. The data on food neophobia were analysed according to Laureati and colleagues [8]. In particular, a neophobia score ranging from 8 to 40 was computed for each child (scores from neophilic items 1, 4, 5, and 8 were reversed), where high scores indicated higher food neophobia. The ICFNS distribution normality was checked using the Shapiro–Wilk test (*p* < 0.05). The frequency distribution of FN scores was calculated, and respondents were divided into three groups according to their FN level (low, medium, and high) using quartile values of the distribution. Chi-square tests (*p* <0.05), followed by significance by cell (Fisher’s exact test), were applied to assess comparability across groups with different FN scores for different variables: gender, NUTS areas, frequency of eating in the canteen, choice of emotions and selection of attributes describing the mealtime (in the two latter cases, occurrences were expressed as percentages) (*p* < 0.05). A two-way ANOVA was run to assess the effect of the FN group on the self-reported liking for 7 food categories in the canteen (fixed factors: FN group, food category, FN group* food category). Similarly, separate one-way ANOVAs were run to assess the effect of the FN group, respectively, on the following variables: pleasure of eating at home, pleasure of eating in the canteen, overall food waste (fixed factors: FN group). All the ANOVA models were followed by the pair comparison Tukey’s test (*p* < 0.05). Letters indicating significant different mean values from Tukey’s test are reported in the text. Analyses were run with XLSstat Marketing 2023.1.6 (Addinsoft, New York, NY, USA, version 2024.3.0).

## 3. Results

### 3.1. Sample Characterisation and Food Neophobia in the Population

The population was balanced for gender (Table 1) and mostly comprised children of Italian nationality. Children were aged 7–11 years (y.o.), with a mean and median age of 9. Half of the children lived in central Italy (52%), and 44% lived in the northern regions of Italy. Most children ate in the canteen (57%); of these, 38% ate in the canteen every day, 14% did so 3–4 times per week, and only 6% ate just 1–2 times per week.

**Figure 1:**
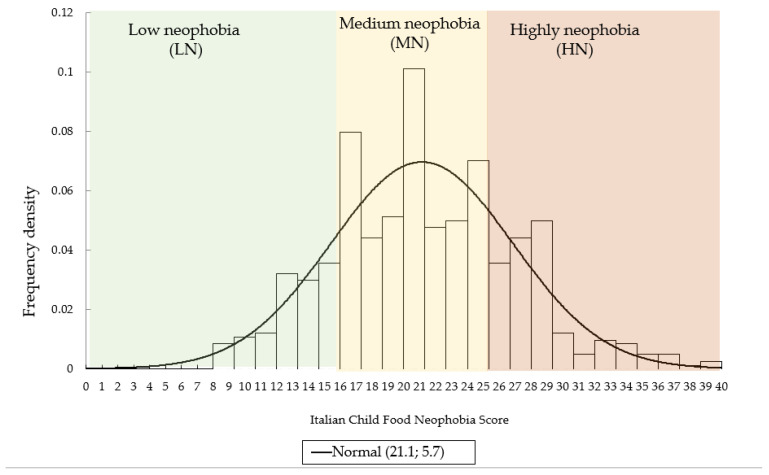
Distribution of the variable ICFNS among children (n = 630).

The ICFNS values in the sample ranged from 8 to 39, and the mean and median values were 21. The first and third quartile values of the ICFNS were 17 and 25, respectively, identifying three groups: low neophobia (LN ≤ 16, n = 135, 21.4%), medium neophobia (MN 17-25, n = 346, 54.9%), and high neophobia (HN ≥ 25, n = 149, 23.7%). The variable ICFNS tended towards a normal distribution (Figure 1), but the test was not significant (W (630) = 1.0, *p* < 0.05).

Groups with different FN levels did not significantly differ (*p* > 0.05) by gender composition or by the distribution of geographical NUTS areas. However, they differed in the frequency of eating in the canteen (X^2^ observed = 17.1; X^2^ critical = 12.6; DF = 6), with HN having significantly fewer children than expected attending every day (27%), while LN had significantly more children than expected attending every day (48%).

### 3.2. Effect of Children’s Food Neophobia on Liking for Food, Pleasure of Eating in the Canteen, and Food Waste

**Figure 2:**
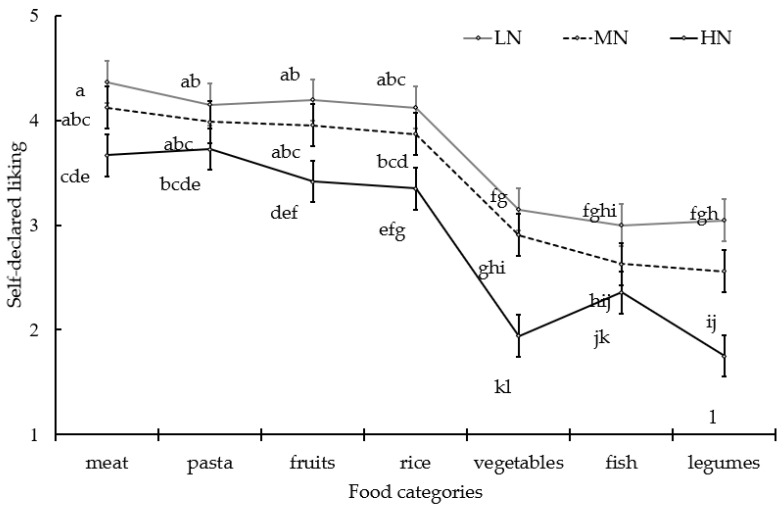
Self-reported liking values for seven food categories given by groups of children (n = 630) with different ICFNS. Note: different letters indicate different mean values form Tukey’s test (*p* < 0.05). LN = low neophobia; MN = medium neophobia; HN = high neophobia.

A significant effect of the food category was found on self-reported liking (F = 171.9, *p* < 0.001, with standard error of the means = 0.05). Meat (4.07a), pasta (3.96ab), and fruits (3.88ab) were the most liked items, with rice having an intermediate mean liking value (3.80b), and vegetables (2.73c), fish (2.64cd), and legumes being the least liked (2.47d). As expected, a strong effect of the FN group was found on self-reported liking (F = 117.4, *p* < 0.001, with standard error of the means = 0.05). The LN group had the highest mean self-reported liking (3.72a) compared to the HN group (2.89c); the MN group had an intermediate value (3.43b). The significant interaction in the FN group*food category (F = 3.1, *p* = 0.05) showed that the LN group had a significantly higher self-reported liking than the HN group for every item and that the maximum difference in liking values was for vegetables and legumes, generally being the mostly disliked foods (Figure 2).

**Figure 3:**
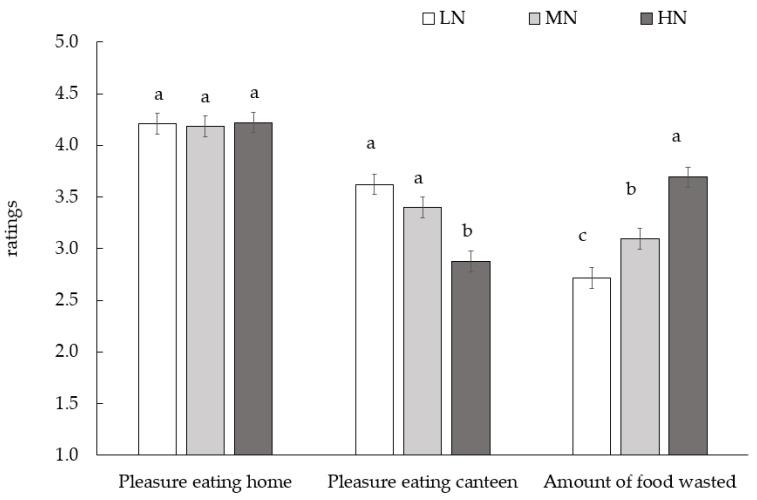
Self-reported pleasure in eating at home, pleasure in eating in the canteen, and amount of food wasted by children (n = 630) with different level of food neophobia. Note: different letters indicate different mean values form Tukey’s test (*p* < 0.05). LN = low neophobia; MN = medium neophobia; high neophobia = HN.

While all groups showed high levels of pleasure in eating at home without differences (*p* > 0.05) (Figure 3), FN groups expressed varying levels of the pleasure in eating in the canteen (F = 17.3, *p* < 0.001) and self-reported overall food wasted in the canteen (F = 33.7, *p* < 0.001) (Figure 3). In particular, the HN group had the lowest pleasure in eating in the canteen and the highest self-reported food waste. Their ratings significantly differed from those given by the LN group for both pleasure in eating in the canteen and self-reported food waste. The LN group had the most positive attitude (high enjoyment of the canteen meal and lower plate leftover). For pleasure in eating in the canteen, the mean ratings of the MN group did not significantly differ from the LN group.

### 3.3. Effect of Children’s Food Neophobia on Meal Description and Emotional Status

We observed a general prevalence of positive emotions over negative emotions. The choice of emotions was significantly influenced by the FN group (X^2^ observed = 73.5, X^2^ critical = 26.3, DF = 16, *p* < 0.05) (Table 2). The LN group selected emotions with negative valence (disgusted, sad) and the neutral emoticon a lower number of times than expected, while the HN group showed the opposite trend in selecting negative emotions (angry, disgusted, sad) and selected the neutral emoticon a higher number of times than expected. Moreover, the LN group was more positive and selected the positive emotions, enthusiastic and full of energy, a higher number of times than expected, which have a high arousal. The HN group instead exhibited a significantly opposite trend compared to the LN group for these positive emotions.

Regarding mealtime perception, the choice of attributes describing the mealtime was significantly influenced by the FN group (X^2^ observed = 38.3; X^2^ critical = 33.9; DF = 22; *p* < 0.05) (Table 3). In particular, the LN group described the meal in the canteen as being significantly less boring than expected, while the HN group perceived it as being more uncomfortable and boring than expected.

## 4. Discussion

### 4.1. Food Neophobia with Respect to Food Familiarity

The mean value of FN that we obtained was in line with a recent cross-cultural study conducted with children 9–12 aged in five European countries (Finland, Italy, Spain, Sweden, and UK) [24].

The fact that the LN group showed the lowest frequency of eating in the canteen was expected, since it is known that exposure towards a situation/environment/food reduces reluctance towards it. In fact, the frequency of consuming a food increases the familiarity towards it, and children with low food neophobia are typically familiar with a larger number of foods than those with high FN [9]. This was also specifically documented for the frequency of fruit and vegetable consumption, which was negatively correlated with FN among British children [22].

This study examined classes of foods that are familiar in Italian cuisine and Italian culture (pasta, rice, vegetables, beans, fruits, meat, fish). The level of neophobia, therefore, did not refer to novel food as in other cases (e.g., ethnic or innovative) [26] but rather to the intrinsic dislike of foods that can be considered familiar in the Italian context. This was particularly true in those foods that are well known as being generally disliked by children, such as legumes [27] and vegetables [28]. Our findings were in agreement with previous results of a large-scale study on the Italian adult population (the “Italian Taste project”), showing that FN encompasses the rejection of familiar food and beverages in adults [29]. A limitation of this study is that we did not present any real food to children; we instead assumed that children were able to appropriately recall them and provide judgements. Despite the use of validated tools, in our case, recalling items (food, contexts, etc.) might have been too complex for children, since it required a level of cognitive effort that may have caused a decrease in the reliability of their answers. However, studying the response for real food in real tasting conditions (i.e., during lunch in school canteens) would have had several practical difficulties that would probably have slowed down or largely reduced the possibility of reaching a high number of participating schools and, thus, of respondents. Future studies should document the relationship we found in the context of real food consumption.

### 4.2. Food Neophobia Among Children with Respect to Liking and Food Waste

Our results are in agreement with an early study in school canteens of the Municipality of Milan, finding that starchy first courses (like pasta and risotto) were generally well liked among children [30]. Moreover, our results also agree with previous studies showing that highly neophobic children eat less fruit and vegetables than their less neophobic peers [22,24]. This is a consolidated result in the literature and was also confirmed in the large-scale “Italian Taste project”, showing that reported liking was significantly lower for the HN than LN group, in particular for those vegetables and beverages characterised by high levels of warning stimuli (i.e., bitterness, sourness, astringency) [29]. In general, we attribute the lower disliking of food in highly neophobic children to different factors, which may also include physiological aspects. For example, according to the literature, high food neophobia was associated with a higher lingual tactile acuity in a group of 6–13-year-old children (measured with the finest Von Frey filament) [23]. Higher sensitivity could, therefore, partially explain the higher rate of rejection for disliked taste or sensory sensations characterising critical food categories like vegetables and legumes. However, we did not have the chance to estimate the oral acuity, so this remains a hypothesis.

On the other hand, in our case, the generally lower liking found among the HN group could have been due to the lower exposure to food in the canteen environment (lower frequency of eating in the canteen). A practical recommendation related to this could be to adopt strategies and actions to increase the exposure of children to the school canteen to reduce FN.

To the best of our knowledge, while food waste has been recently tackled in the literature in families with children [31] or in school canteens [20,32], the specific relationship between FN and wasted food has not yet been documented either among adults or children. However, we expected a positive relationship, which was confirmed in this study, due to the fact that highly neophobic people generally show a higher selectivity towards food and that FN partially overlaps with pickiness [33], both factors possibly translating into higher FN and a higher level of leftover food on plates. In this sense, our study contributes by increasing the knowledge of FN and its effect on food behaviours, being among the first to document a direct link between FN and food waste.

### 4.3. Food Neophobia Among Children with Respect to Emotions and Meal Description

In general, the prevalence of positive emotions over negative emotions for eating in the school canteen was expected, since it is known that food is generally and mostly associated with positive emotions [34], a finding previously observed among pre-adolescents [18]. According to a recent review, studies on children’s perspectives on meals in childcare in early years demonstrate that children predominantly judge food based on taste and positive emotions [35]. Similarly, a participatory study with Swedish children observed that positive emotions were an additional reason, together with the preference for taste, to prefer a specific type of food, such as Christmas rice pudding [36]. In this sense, our results are, therefore, in full agreement with the literature.

Our key finding was that the LN group had an overall better positive mood compared with the HN group, particularly the reported high frequencies of positive emotions with high arousal. The positive emotional status was also probably linked to the fact that children with LN described the meal in the canteen as being more comfortable, and this positive aspect is related to a positive emotional status. In this sense, the perception of the school canteen, meant as a set of perceptive inputs (sounds, colours, layout of furnishings, smells, etc.), can indeed also be seen as an environment that can modulate children’s emotional status and thus finally mediate their eating behaviour.

### 4.4. Food Neophobia Among Children with Respect to Food Procurement Policies

In technical regulations for the procurement of food for school canteens, neophobia is conspicuously absent. The prevailing Italian standards CAM—Minimum Environmental Criteria [37] (approved with the Ministerial Decree 65, 10 March 2020)—and their accompanying report place significant emphasis on the environmental implications of supply chains, the diversity of food supplies, and the quality of food offered. However, these standards do not address aspects that may impact FN, such as the degree of pleasantness of and (un)familiarity with the food offered, environmental aspects in the food service that increase the palatability of food, and aspects that may improve the space/context in which the school meal occurs, such as the possibility of socialisation, etc. Consequently, the legal framework imposes upon operators the onerous task of procuring food, the rationale for which is not always clear [38], without necessarily generating tangible nutritional and educational benefits.

Currently, there is a lack of awareness in Italian public policies about the impact of the neophobic condition of primary school children, combined with a lack of criteria to estimate the use of practices that could mitigate the effect of FN. Consequently, children may not consume costly and difficult-to-prepare meals despite their nutritional value and thus significantly contribute to the amount of food waste (see Figure 3 for details). It is necessary to integrate approval and food education choices, encompassing both children and families, to avert the potential for a fragmented approach. This is particularly salient in the context of school nutrition, where a multitude of stakeholders, including the government, collective catering services, educational institutions, and families, aim to improve the diet of children by enhancing the quality of school meals. However, their efforts might prove futile without a cohesive, unified strategy based on food education.

### 4.5. Limitations and Future Perspectives

One limitation of our study was the unbalanced sample composition, which saw most children coming from central Italy, and the fact that we did not consider the effect of geographical provenience on the responses. Indeed, several regional differences in the gastronomic traditions exist in Italy based on historic, sociological, and epidemiological aspects, translating into differences in culinary praxis and the prevalence of flavours and ingredients [39]. However, we made efforts to develop an experimental design by envisaging classes of products (pasta; rice; vegetables; beans—peas, lentils, etc.; fruits; meat; fish) highly relevant to any region, independently of the children’s provenience.

Secondly, the length of our questionnaire may be considered a weak point. In fact, questionnaires targeted towards children often comprise a relatively lower number of questions [21], and, as recently reviewed for adults, a lengthy questionnaire can induce boredom, foster a lack of attention, and negatively affect completion rates [40]. This may, therefore, have partially negatively affected the validity of our data. However, the degree of complexity of our experimental (envisaging the relationship between different variables) made it necessary to incorporate a high number of questions and thus extend the survey’s length. Moreover, when developing questionnaires for children, particular care should be taken in establishing age-appropriate protocols adopting simplified questionnaires with adequate scales and using child-friendly vocabulary [41]. In this sense, we made significant efforts in both aspects by adopting validated scales suited to the age range considered and using simpler vocabulary.

As a third point, another weakness resides in having collected self-reported data. It is known that in self-reported (not measured) conditions, children may experience some difficulties in recalling situations (that is, in accurately remembering what they ate or how much they ate, especially over longer periods) [42], they may have cognitive limitations that reduce their ability to understand and respond to questions accurately [21], they may suffer from acquiescence bias (they tend to agree with whatever the adult says or answer “yes” to questions regardless of the truth) [43], or they might suffer from social desirability bias (they may answer in a way they think will make them look good or please the adult) [44]. However, it is important to mention that these limitations apply to all studies dealing with self-reported data.

Future perspectives envisage the development of observational measures in real contexts (e.g., school canteens), the expansion of the experimental with biometric measures, and the extension of the observations to other interesting study targets, for example, teenagers.

## 5. Conclusions

Our study addressed the topic of FN in children by considering the relationship between this relevant and highly studied personality trait with a group of variables (pleasure in eating in the canteen, liking for different food categories, emotional responses, meal description, and food waste) that have so far mostly been treated separately. In general, this study contributes by broadening the picture of FN in children. In particular, we highlighted that those children with low FN showed a higher self-reported liking for most food categories eaten in the school canteen, and they show a more positive emotional status (particularly regarding positive emotions with high arousal, i.e., enthusiastic and full of energy) and meal description perception (the meal is comfortable) that translate into lower self-reported food waste in the canteen.

Working on and improving elements of the school canteen as a socialising space (referring to the possibility/degree of comfort of the furniture and service flow, the degree of noise/sounds, the presence of other people, etc.) can modulate children’s emotional responses and finally the eating behaviour of children towards improving food waste.

Our findings may be of interest to experts working in the field of education, nutrition, and regulations (concerning the legal aspects and guidelines of school management), since they suggest that interventions should consider the emotional status of children eating in the canteen to nudge them towards virtuous behaviour (e.g., reducing food waste).

## Figures and Tables

**Table 1:** Characteristics of the child population (n = 630).

Variable	Categories	N	%
Gender	F	336	53
	M	294	47
Nationality	Italian	584	93
	Not Italian	43	7
	Did not answer	3	0
Class	3rd	184	29
	4th	230	37
	5th	216	34
Geographical area ^1^	Northwest (NW)	159	25
	Northeast (NE)	120	19
	Centre (C)	325	52
	South (S)	26	4
School city	Cinisello Balsamo, Milan (NW)	73	12
	Milan (NW)	86	14
	Legnago, Verona (NE)	40	6
	Formigine, Modena (NE)	80	13
	Florence (C)	88	14
	Perugia (C)	68	11
	Ladispoli, Rome (C)	42	7
	San Marcello, Ancona (C)	127	20
	Salerno (S)	26	4
Frequency of eating at canteen	1–2 times per week	38	6
	3–4 times per week	87	14
	Every day	237	38
	Did not answer	268	43

^1^ NUTS (classification of territorial units for statistics) area from Regulation (EC) No 1059/2003.

**Table 2:** Frequencies of emotions choices (%) across groups of children (n = 630) with different food neophobia level.

Valence	Emotions *	LN (n = 135)		MN (n = 346)		HN (n = 149)	
**Negative**	**Angry (arrabbiato)**	**4**	**<**	6	<	**13**	**>**
	Disgusted (disgustato)	**21**	**<**	21	<	**40**	**>**
	Annoyed (infastidito)	8	<	11	>	15	>
	Sad (triste)	**6**	**<**	8	<	**19**	**>**
Neutral	Neutral (neutro)	**21**	**<**	35	>	**43**	**>**
Positive	Satisfied (soddisfatto)	54	>	44	>	**31**	**<**
	Happy (felice)	61	>	53	>	40	<
	Enthusiastic (entusiasta)	**42**	**>**	23	<	**14**	**<**
	Full of energy (pieno di energia)	**67**	**>**	40	<	**27**	**<**

Note: ‘<’ and ‘>’ symbols were obtained from Fisher’s exact test for significance by cell, with values in bold being significant (α = 0.05). LN = low neophobia; MN = medium neophobia; HN = high neophobia. * The original Italian translation of the emotions are in brackets.

**Table 3:** Frequencies of mealtime description choices (%) across groups of children (n = 630) with different food neophobia levels.

Variable	Categories	LN (n = 135)		MN (n = 346)		HN (n = 149)	
**Meal length**	**Short**	**47**	<	45	<	48	>
	Long	53	>	55	>	52	<
Presence of noise	Noisy	87	>	86	<	89	>
	Silent	13	<	14	>	11	<
Meal comfort	Comfortable	78	>	79	>	**59**	**<**
	Uncomfortable	22	<	21	<	**41**	**>**
Place crowding	Crowded	81	>	78	<	78	<
	Empty	19	<	22	>	22	>
Overall mealtime fun	Funny	82	>	78	>	**56**	**<**
	Boring	**18**	**<**	22	<	**44**	**>**
Possibility of movement	Not free to move	48	<	45	<	60	>
	Free to move	52	>	55	>	40	<

Note: ‘<’ and ‘>’ symbols were obtained from Fisher’s exact test for significance by cell, with values in bold being significant (α = 0.05).

## Data Availability

The raw data supporting the conclusions of this article will be made available by the authors on request.
